# New perspectives for neutron imaging through advanced event-mode data acquisition

**DOI:** 10.1038/s41598-021-00822-5

**Published:** 2021-11-01

**Authors:** A. S. Losko, Y. Han, B. Schillinger, A. Tartaglione, M. Morgano, M. Strobl, J. Long, A. S. Tremsin, M. Schulz

**Affiliations:** 1 Forschungs-Neutronenquelle Heinz Maier-Leibnitz, 85748 Garching, Germany; 2 Paul Scherrer Institute, 5232 Villigen, Switzerland; 3 Amsterdam Scientific Instruments, 1098XG Amsterdam, The Netherlands; 4 Space Sciences Laboratory, University of California at Berkeley, Berkeley, CA 94720 USA

**Keywords:** Imaging and sensing, Imaging techniques, Experimental particle physics, Imaging techniques

## Abstract

Imaging using scintillators is a widespread and cost-effective approach in radiography. While different types of scintillator and sensor configurations exist, it can be stated that the detection efficiency and resolution of a scintillator-based system strongly depend on the scintillator material and its thickness. Recently developed event-driven detectors are capable of registering spots of light emitted by the scintillator after a particle interaction, allowing to reconstruct the Center-of-Mass of the interaction within the scintillator. This results in a more precise location of the event and therefore provides a pathway to overcome the scintillator thickness limitation and increase the effective spatial resolution of the system. Utilizing this principle, we present a detector capable of Time-of-Flight imaging with an adjustable field-of-view, ad-hoc binning and re-binning of data based on the requirements of the experiment including the possibility of particle discrimination via the analysis of the event shape in space and time. It is considered that this novel concept might replace regular cameras in neutron imaging detectors as it provides superior detection capabilities with the most recent results providing an increase by a factor 3 in image resolution and an increase by up to a factor of 7.5 in signal-to-noise for thermal neutron imaging.

## Introduction

Over the past decades, digital cameras have replaced their analog counterparts and despite the advancement in smaller pixel-pitch, faster read-out and lower read-out noise, the principles of how digital cameras record images have not changed much. For that matter, there are two main types of digital image sensors, namely the charge-coupled device (CCD)^1^ and the complementary metal–oxide–semiconductor (CMOS) sensor^2^, with the main difference between the two being the read-out. For this work, the sequential (or individual) pixel read-out of most CMOS sensors is crucial, because it enables the recently developed Timepix3 chip^3^ to register the Time-of-Arrival (ToA) and the intensity of light in each pixel (through the Time-over-Threshold (ToT) method) for each event simultaneously and sparsely with up to 80 Mhits/s. Here, a “hit” or “event” is defined as any one of the 256 × 256 pixels composing the chip (with a pitch of 55 μm and a nominal time-resolution of 1.5625 ns) being activated, i.e. having some charge over a certain threshold being deposited on it at a specific ToA for a specific ToT. While the Timepix3 chip requires a charge above ~ 500–1000 electrons, and thus has a low sensitivity to visible light photons, Amsterdam Scientific Instruments (ASI) has developed the TPX3Cam^4–6^, a light sensitivity-enhanced silicon sensor based Timepix3 camera system that can image and time-stamp light flashes of more than 10^3^ photons with high quantum efficiency (> 90%) in the 400 to 1000 nm wavelength-range^7^. It is suited for application to imaging of electrons, ions or even single photons. For the latter case, the threshold of 10^3^ photons is certainly too high for the TPX3Cam to detect a single photon directly. To overcome this limit, an image intensifier is needed to amplify light that would otherwise not reach the threshold. This applies in particular for example to light coming off a neutron scintillator screen, as it is the case for this work. With high enough amplification (> > 10^3^), even single photons can be detected^5,6^. Utilizing this capability, we have developed a new approach for particle counting using scintillators, changing the principles of how the radiographs are recorded. This is achieved with parameters such as spatial and time resolution being defined after the measurement and not directly defined *ab-initio* by the pixel-size or the timing resolution of the system, but more so by the capability of detecting clusters of photons emitted by the scintillator after a particle interaction via the coincidence of many pixels activated over time.

Using simple Center of Mass (CoM) algorithms for single particle interactions and detection of the consequently emitted photons, the position of the particle’s interaction can be determined more accurately. This bears analogy to a similar digital imaging principle to that of the already applied principle in the determination of the position of stars in astronomy since 1980^8^. In a sense, each interaction on the scintillator material creates a “star-like” signature. Thus, the idea to use CoM for improving position accuracy in digital imaging is basically as old as digital imaging itself. Nonetheless, applying a CoM to a single object, *versus* running such an analysis on single photon events for imaging purposes efficiently requires a detector technology that has shifted from a frame-based to an event-based system, such as the Timepix3 chip, as well as the necessary computational power to perform the analysis in real-time.

First studies on this concept applied to neutron imaging were reported on a frame-based imaging system^9^. However, the reported measurements faced limitations in acquisition dead-time and data processing time due to the slow read-out and noisy data inherent to the camera system in use for that study. Moreover, results were produced from post processed data only. While real-time single-event data-acquisition for neutron imaging was demonstrated on a Timepix quad detector combined with a neutron sensitive Microchannel-Plate (MCP)^10^ with successful application to energy-resolved neutron imaging^11^, the detector system in these applications has a fixed FoV based on the active area of 28 × 28 mm^2^. For the work presented here, it was crucial to find a solution that would allow for a variable FoV as it is essential state-of-the-art in neutron imaging today. Therefore, using a scintillator-based light collection system has the advantage compared to an MCP detector^10^ to use optics for adjustment of the FoV, which is crucial for imaging of different length scales and objects of different sizes, a requirement that applies to essentially all neutron imaging beam-lines.

Aside from the FoV, additional focus of this study was to achieve as high spatial resolution as possible by taking advantage of the event-based data-acquisition. A recent example of using a TPX3Cam optical camera^5^ showed that it is possible to improve the optical resolution of the camera beyond the pixel size of the sensor via CoM photon detection. An effective resolution equal to 1/5 of the pixel size of the sensor with a temporal resolution of better than 5 ns was demonstrated. In comparison to the described setup in that work, here the target for the camera concept is neutron imaging and, thus, essentially detecting the light a scintillator screen emits after a neutron is absorbed. Hence, the setup is very similar in principle with the caveat of light emission from the scintillator being time-dependent and generally based on several exponential decay functions^12–14^. Therefore, in this work, we go beyond the identification of single photons for optical resolution improvement to detecting clusters of photons that can be attributed to single neutron events.

In similar efforts at the China Spallation Neutron Source (CSNS), the ToA capability of the TPX3Cam was utilized to perform energy resolved neutron imaging at small FoVs of 5.4 × 5.4 mm^2^^15^. The approach for neutron detection in that work is similar to that of the technique that was applied to the MCP detector^10^ for energy-resolved neutron imaging^11^ as discussed previously, in that a single CoM algorithm to events observed on the detector is described. However, the crucial difference between the two detector concepts is that for the MCP detector^10^ the neutron capture produces a single electron avalanche (initiated by an alpha or ^7^Li particle) in the borated MCP pores, providing a prompt cascade of electrons that is detected by the Timepix sensor. In contrast, the TPX3Cam in combination with an image intensifier (chevron configuration) and scintillator detects the cascade of electrons triggered by single photons amplified by an MCP intensifier, whereby for each captured neutron hundreds to thousands of photons, depending on the scintillator, are emitted over time, with only a fraction of these photons reaching the image intensifier based on the light-collection efficiency. Without a mechanism to address this time depended process of light emission of the scintillator, the TPX3Cam does not provide significant advantage compared to e.g. low cost fast CMOS cameras for ToF neutron imaging^16^. However, in this work we show that by carefully adjusting the light collection parameters of the setup, it is possible to detect individual neutron events with spatial and time resolution using a two-stage approach, that is photon detection via CoM of single pixels and neutron detection via detection of clusters of photons. The added complexity in turn provides a solution to the afterglow problem for scintillators^13^ and potentially enables discrimination of events in routine imaging measurements, where e.g. neutron and gamma events could be distinguished based on the light-yield decay properties of the interaction in the scintillator. A similar principle to reduce the gamma background for neutron transmission or diffraction measurements is also applied to photo-multiplier tube (PMT) detectors^17–19^, as well as for wavelength shifting fiber detectors deployed at many facilities around the world^20^. However, a major drawback of PMTs or wavelength shifting fiber detectors is that their spatial resolution is very limited, with the highest resolution systems providing only ~ 100 μm spatial resolution and similar to the MCP detector^10^ they have fixed FoVs, rendering them unusable for most imaging applications. Another benefit of using the TPX3Cam is that not only does it provide access to the event shape in time, but it also provides detailed shape information in space, with the capability of using optical lenses to focus or defocus the events. This adds the flexibility of optically enlarging the spread of the photons, which is useful if for example the event is smaller than the pixel in size or if the local photon count-rate is too high for a single neutron event. At the same time, for a setup with a poor focal depth, the CoM algorithm will still allow to determine the origin of the event since the spread of the photons will be concentric around the origin. For regular cameras, the depth of field can only be increased by reducing the aperture on the lens, consequently also reducing the light collection efficiency, a limitation that is overcome with the technique presented here.

The first measurements with neutrons for this work were conducted at the Beamline for neutron Optics and other Approaches (BOA)^21^ at the Paul Scherrer Institute (PSI) in Switzerland, with data collected in “regular” white beam neutron imaging mode and Time-of-Flight (ToF) transmission mode enabled by a disk chopper installed in the beam to determine the scintillator response and to characterize the system. Furthermore, Bragg-Edge imaging^22,23^ and dynamic response measurements of different neutron scintillator materials were performed, but will be reported in more detail at a later stage elsewhere.

This first report focuses on the concept of the event-based image reconstruction. In particular, we demonstrate a two-stage process in the analysis, that is in a first stage: photon imaging mode — identifying photons by grouping pixels corresponding to one photon, and in a second stage: neutron imaging mode — identifying neutrons by grouping photons corresponding to one neutron. In particular, the second stage, neutron imaging mode, is fundamentally crucial for overcoming the limitations of conventional imaging techniques, making the here presented imaging concept a very powerful tool for radiography using, but not limited to, neutrons.

## Event-mode imaging setup for neutrons

**Figure 1:**
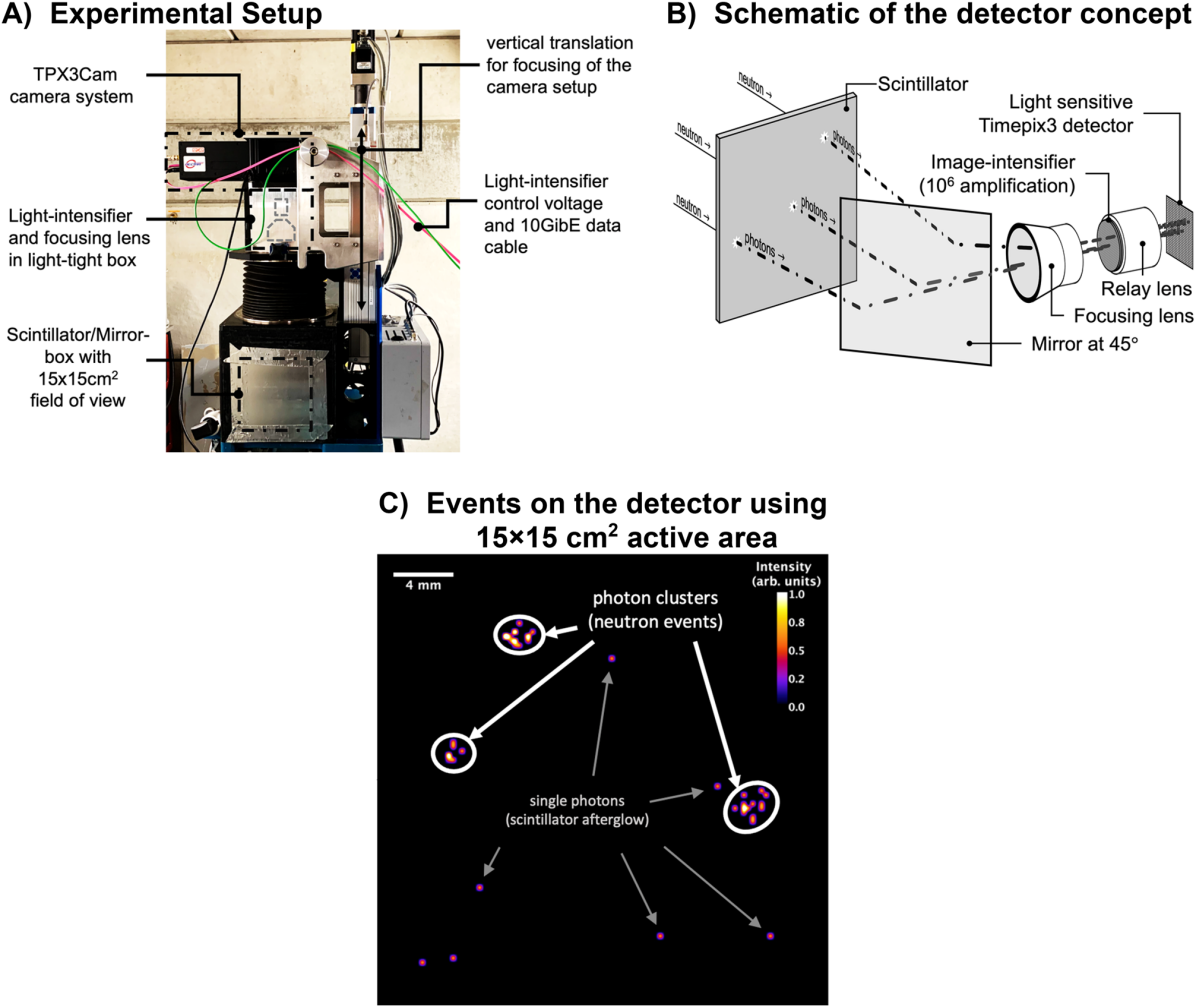
Photograph of the experimental setup at the BOA beam-line in (**A**). Schematic of the detector concept (not to scale) in (**B**). Single photon-events measured with Timepix3 detector in C highlighting scintillator afterglow and neutron events.

The components of the system, listed sequentially viewed from the source (see Fig. 1), are a ^6^LiF:ZnS scintillator (> 95% ^6^Li enrichment) for thermal neutrons positioned in the direct beam, a mirror positioned at 45° relative to the scintillator to reflect photons coming off the scintillator out of the direct beam path, a focusing lens to adjust the focus onto the scintillator surface, an MCP image intensifier (with P47 phosphor, up to 10^6^ amplification, 6 μm MCP pore size, 8 μm pitch, two stack MCP mounted in chevron configuration) including a relay lens, and the ASI optical camera TPX3Cam based on a Timepix3 readout chip. It should be noted that the principle experimental setup for the measurements is identical to that of “regular” neutron imaging detectors using scintillators^24^, apart from the camera system. Moreover, for the measurements, the BOA beamline was essentially operated in its standard configuration and only the camera replaced by the intensified TPX3Cam camera. A photograph of the setup at the BOA beamline and a schematic of the setup are shown in Fig. 1A,B, respectively.

As it is crucial for measurements using lenses that they are properly focused, the relay lens of the intensifier was focused using a low flux of photons hitting the image intensifier, such that the average size for a single photon on the detector resulted in the activation of ~ 3** × **3 pixels (average per photon event). The second focusing lens (between mirror and light intensifier, see Fig. 1B) was adjusted using short acquisitions while observing and minimizing the radius of clusters of photons on the scintillator (neutron events) as illustrated in Fig. 1C. Inspecting the events, the clusters of photons can be identified by the naked eye. Additionally, single photons without a direct correlation in position or time with other photon events were identified and as will be shown in the following section can be mostly attributed to the scintillator afterglow. These un-correlated photons were expected since each neutron interaction excites multiple photons in the scintillator (> > 3) within a short time-frame (~ 1 ms). In order to characterize the spatial and temporal parameters of the events, the ToF of each registered photon was also measured to correlate the spatial information of the photons with respect to their timing for each neutron interaction.

### Neutron time-of-flight measurements of the detector response

In a first step to entangle the spatial and temporal information of the photons, measurements were performed at the BOA beamline with the instrument operating in ToF mode^21^. Data with a large FoV (15** × **15 cm^2^) and a 50 μm ^6^LiF:ZnS scintillator were acquired using a single disc chopper. The chopper disc to detector distance was set to 6.45 m. The wavelength resolution, $\Delta \lambda /\lambda$,in this configuration resulted in ~ 1% (at 4 Å), calculated using the chopper disc radius of 200 mm, the slit of 1.8 mm with a length of 30 mm from the outer diameter of the disc and the 25 Hz rotation frequency.

**Figure 2:**
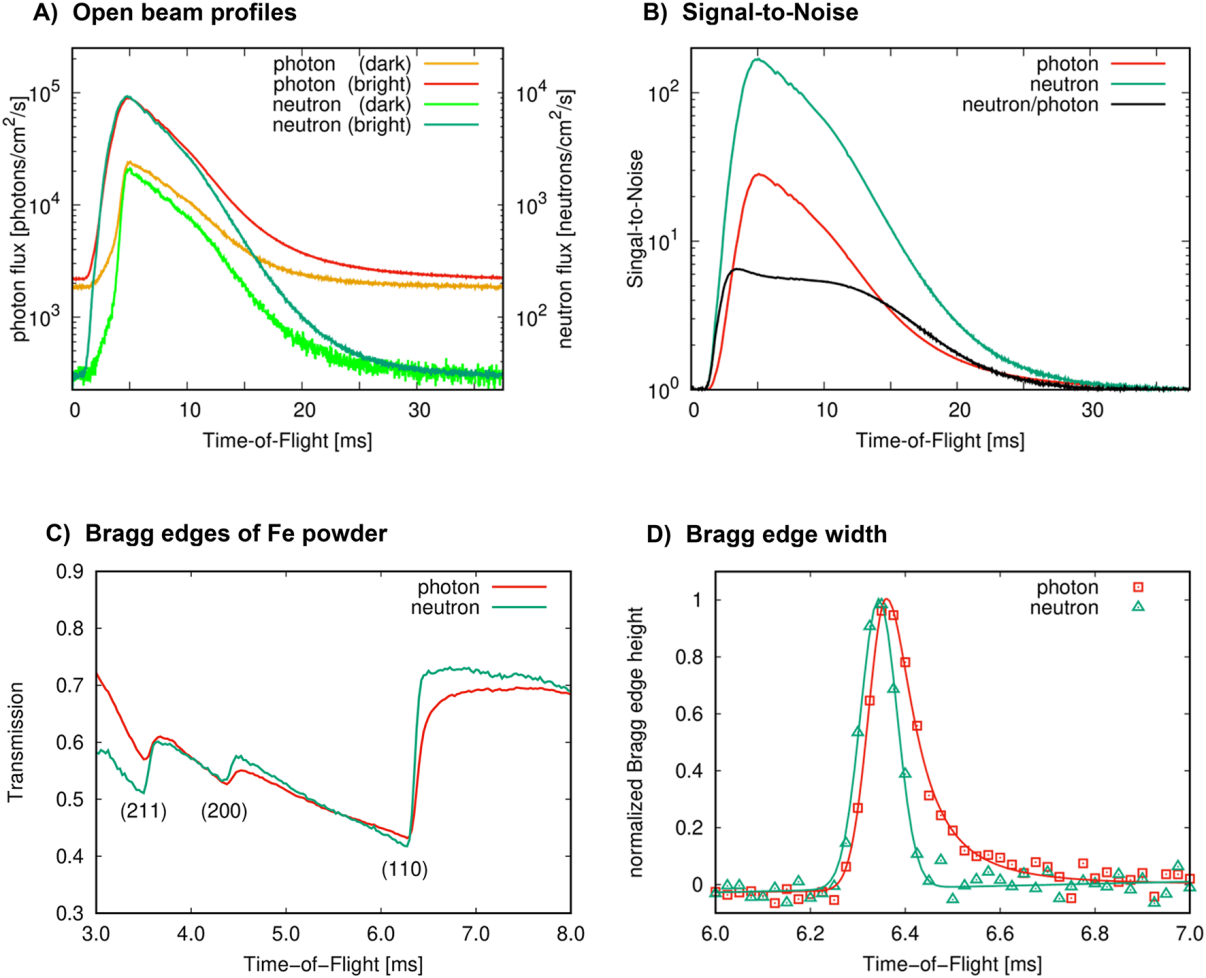
Detected photon and neutron flux in event-mode in (**A**), with photon intensities plotted on the left y-axis and neutron intensities plotted on the right y-axis. In the title, “dark” corresponds to regions of lower intensities and “bright” to regions of higher intensities of the incident beam of selected active detector areas. Signal-to-noise in photon and neutron event-modes averaged over the entire FoV in (**B**). Bragg edge profiles of 2 cm of iron powder measured in photon and neutron event-mode in (**C**). Derivative of the Bragg edge profiles with the peak height normalized to unity for comparison of the time response in photon and neutron event-mode in (**D**).

The number of interactions on the scintillator was measured in this configuration in photon and neutron event-mode for a total acquisition time of 20.4 h, which is shown in Fig. 2A. It should be noted that the term “flux” in Fig. 2 does not correspond to the actual flux of photons or neutrons at BOA in the configuration as shown in Fig. 1, but is correlated to the number of detected events in photon or neutron event-mode. For that matter, for each detected neutron, about ten photons were detected on average by the TPX3Cam.

**Figure 3:**
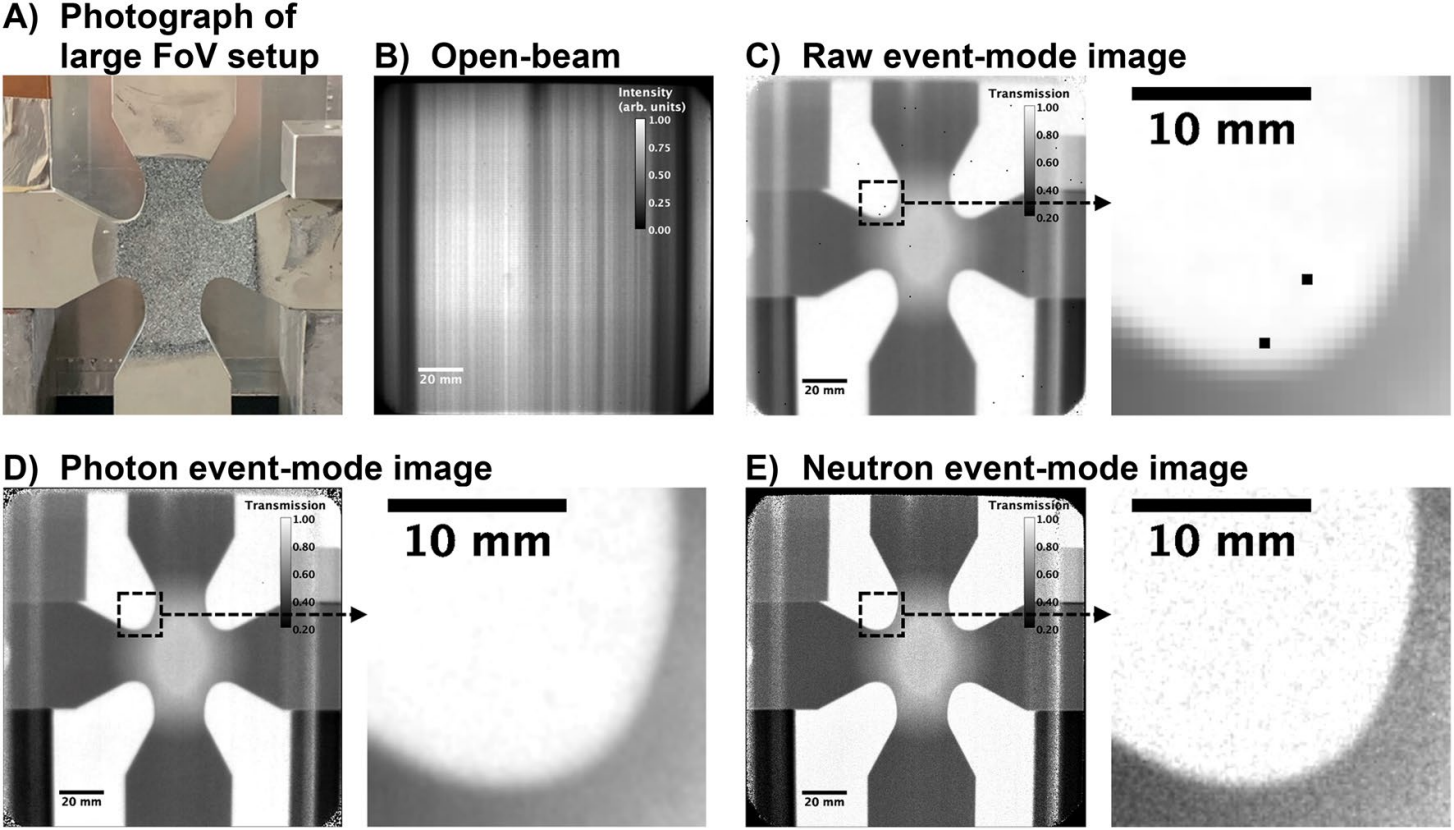
Photograph of various objects positioned at 10 cm distance to the scintillator in (**A**) (steel cross in the center, lead bricks on the bottom left and right, copper sheets on the top left and aluminum cube on the top right). Open-beam using CoM neutron events at sub-pixel resolution of ¼ pixel-pitch in (**B**). Normalized radiographs, including an enlarged section of ~ 2 × 2 cm^2^, produced from: raw event data using the native resolution of the chip in (**C**); CoM photon events at sub-pixel resolution of ½ pixel-pitch in (**D**); CoM neutron events at sub-pixel resolution of ¼ pixel-pitch in (**E**).

Two different areas on the detector with lower and higher incident intensities, denominated in Fig. 2A by “dark” and “bright” regions, respectively, were selected for a direct comparison of the detector response under different neutron fluxes. The lower and higher intensity regions result from the inhomogeneity of the incident neutron flux and will be addressed in the following section (see Fig. 3B). For both regions, it can be observed that at a ToF of t ~ 5 ms (or ~ 4.5 Å) the flux reaches its maximum, whereby t > 30 ms (corresponding to wavelengths > 25 Å) the profile flattens. It is assumed that in the flat region the majority of photons corresponds to the long-lived fluorescent emission, also known as scintillator afterglow. Therefore, as a good approximation, single photons t > 30 ms are emitted randomly from the scintillator surface, not contributing to the neutron-related photon clusters as discussed earlier. As a result, with the here presented neutron event-mode, the afterglow can be essentially eliminated via rejection of these events. This can be observed by the significantly lower intensities of neutrons at t > 30 ms relative to the peak of the profile at ~ 5 ms in Fig. 2A. More specifically, Fig. 2A shows that independent of the intensity of the neutron event-based data for bright or dark regions on the detector area (localized higher or lower neutron flux), the measured flux drops to the same value of ~ 30 neutrons/cm^2^/s at t > 30 ms and can be attributed to a constant background. This background is typical for neutron ToF measurements^25^ due to the experimental cave made from concrete filling up with essentially a “neutron gas”, which to a good approximation can be assumed constant throughout the entire ToF range. The photon event-based data in contrast shows a significantly different “stationary” value at t > 30 ms for bright and dark detector regions, resulting from the fluorescent afterglow effect that is dependent on the localized exposure of the scintillator by neutrons, inducing the consequently emitted photons.

Assuming that for t → ∞ the signal on the detector is purely noise, the signal-to-noise (S/N) ratio for the measured photon and neutron event-based fluxes averaged over the entire detector area are shown in Fig. 2B. Comparing the neutron and photon profiles within the brightest regions on the detector, the S/N ratio improved by up to a factor of 7.5, with a S/N ratio of up to 300.

In the same configuration, Bragg edge profiles of 2 cm of iron powder enclosed in an aluminum container with 2 mm wall thickness were measured. The sample was positioned right after the chopper to minimize secondary contributions in the background by the sample with data acquired for ~ 4 h. The profiles measured in photon and neutron event-mode are shown in Fig. 2C. Inspecting the profiles, long tails of the Bragg-edges can be observed in the photon event-mode data when compared to the neutron event-mode data. This can be furthermore illustrated by taking the derivative with respect to time of the profiles as shown in Fig. 2D, whereby the photon event-mode profile shows an asymmetric peak profile (fit with a split Voigt function) and the neutron event-mode profile in contrast shows a symmetric behavior (fit with a single gaussian function). Here, the photon event-mode means that no clustering of photons into single-neutron related response is performed during data analysis. The full width at half maximum for the neutron event-mode profile was determined to be 90.6 ± 1.5 μs. The symmetry of the neutron event-mode profile furthermore shows that the afterglow behavior of the scintillator is removed. Furthermore, in Fig. 2C it can be observed that for the photon event-mode profile the (200) Bragg-edge shows transmission values greater than that of the (211) Bragg-edge as compared to in the neutron event-mode profile, with the neutron event-mode profile consistent with alpha-iron profiles measured using neutron counting detectors reported in the literature^26,27^. This can be explained by the lower S/N of the photon event-mode data as shown in Fig. 2B. As a consequence, more noise in the data artificially increases the transmitted intensities, creating large errors in the computed transmissions values. Eliminating these artifacts, asymmetric Bragg-edge profiles, as well as the error in transmission values as shown in the photon event-mode profile (Fig. 2C,D), highlights the here presented concept for neutron event detection.

For the neutron event-mode data, similar to counting type detectors, errors can be calculated by the square-root of counts. This is an important detail since regular frame-based cameras generally provide grey values and error analysis can only be performed by e.g. inspecting the variation in the data. Therefore, with the here presented principle, counting statistics to the radiographs in neutron event-mode can be applied, overcoming the burden of qualitative images only, as it is the case for regular frame-based cameras. As an example, for the neutron transmission of the Fe powder in Fig. 2C this resulted in 1.27%, 0.95% and 0.81% relative error at 3 ms, 5 ms and 7 ms ToF, respectively.

### Large field-of-view imaging (15 × 15 cm^2^ active area)

Using the setup described in the previous section, several objects were imaged with a 15** × **15 cm^2^ FoV at 6.45 m distance from the chopper to the scintillator and a total acquisition time of 9.27 h. A photograph of the objects, in particular a cruciform steel sample for biaxial tensile load studies, positioned at 10 cm distance from the scintillator is shown in Fig. 3A, while Fig. 3B shows an open beam image taken without sample. Figure 3C–E show normalized neutron radiographs of a sample constructed from different event-modes. Inspecting the radiographs, the photon (Fig. 3D) and neutron (Fig. 3E) event-based images show incrementally improved image qualities when going from raw (Fig. 3C) to photon image reconstruction in a first step and from photon to neutron event image reconstruction in a second step. The improved image quality becomes particularly apparent in the enlarged sections of the images as shown in Fig. 3C–E below the full FoV. Vertical stripes were observed in all radiographs and can be attributed to a very non-uniform incident beam with flux depleted regions that remain as artefacts despite open beam normalization. The non-uniformity is caused by a multi-channel bender neutron optic upstream of the beam exit into the instrument. Through the utilized beam limiting slit at the chopper in the instrument the bender channel structure is imaged, causing a horizontal variation in intensities by more than an order of magnitude within the measured FoV as can be observed in Fig. 3B. Nonetheless, despite the large variations in intensity, most of the detector area was reasonably well normalized.

**Figure 4:**
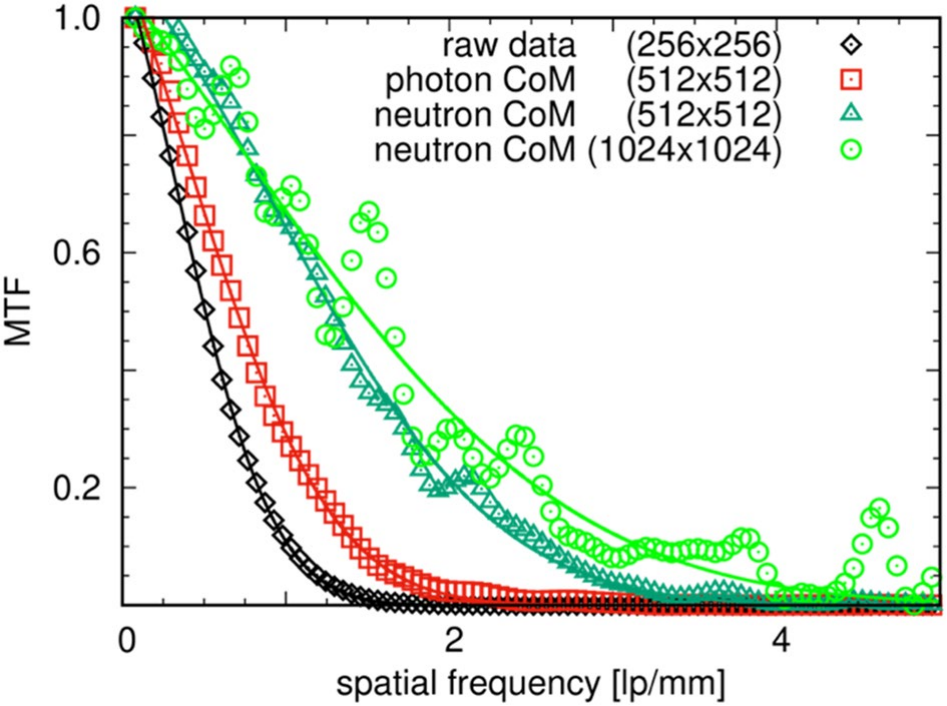
MTF for radiographs of different event-types and sub-pixel pitch for 15** × **15 cm^2^ FoV neutron imaging: for raw event data at the native resolution of the chip; event-based data at sub-pixel resolution by CoM of the photon events at ½ pixel-pitch; CoM of neutron events using ½ pixel-pitch; and CoM of neutron events using ¼ pixel-pitch. Solid lines in the corresponding colors are fitted error functions to the data.

The modulation transfer function (MTF) using the slanted edge method^28^ applied to the slanted edges provided by the steel cruciform (Fig. 3) for the different event-modes was computed and is shown in Fig. 4. Using the raw event-data, the resolution at 50% MTF equates to 0.508 lp/mm or 984 μm. Applying the CoM for individual pixels to detect photon events, the resolution at 50% MTF improved to 0.703 lp/mm or 711 μm using ½ pixel-pitch in the image reconstruction. By furthermore applying the CoM for individual photon events for neutron detection as described earlier, the resolution at 50% MTF improved to 1.289 lp/mm or 388 μm using ½ pixel-pitch in the image reconstruction. While the resolution increased significantly going from raw to photon to neutron event-mode, it can be shown that by furthermore reducing the effective sub-pixel size to ¼ pixel-pitch the resolution continues to increase, with an improved resolution at 50% MTF of 1.466 lp/mm or 341 μm using the neutron event-based data. However, by splitting the events into smaller and smaller bins, the standard deviation for each sub-pixel increases, limiting in this case the practical sub-pixel size to ~ ¼ pixel-pitch at which the MTF can still be computed. The resulting lower statistics for each pixel at higher resolution can also be observed in the increased variation of the MTF. Large deviations of intensities of neighboring pixels in the data can shift the MTF within a specific frequency range, causing the observed variations.

### Small field-of-view imaging (4 × 4 cm^2^ active area)

**Figure 5:**
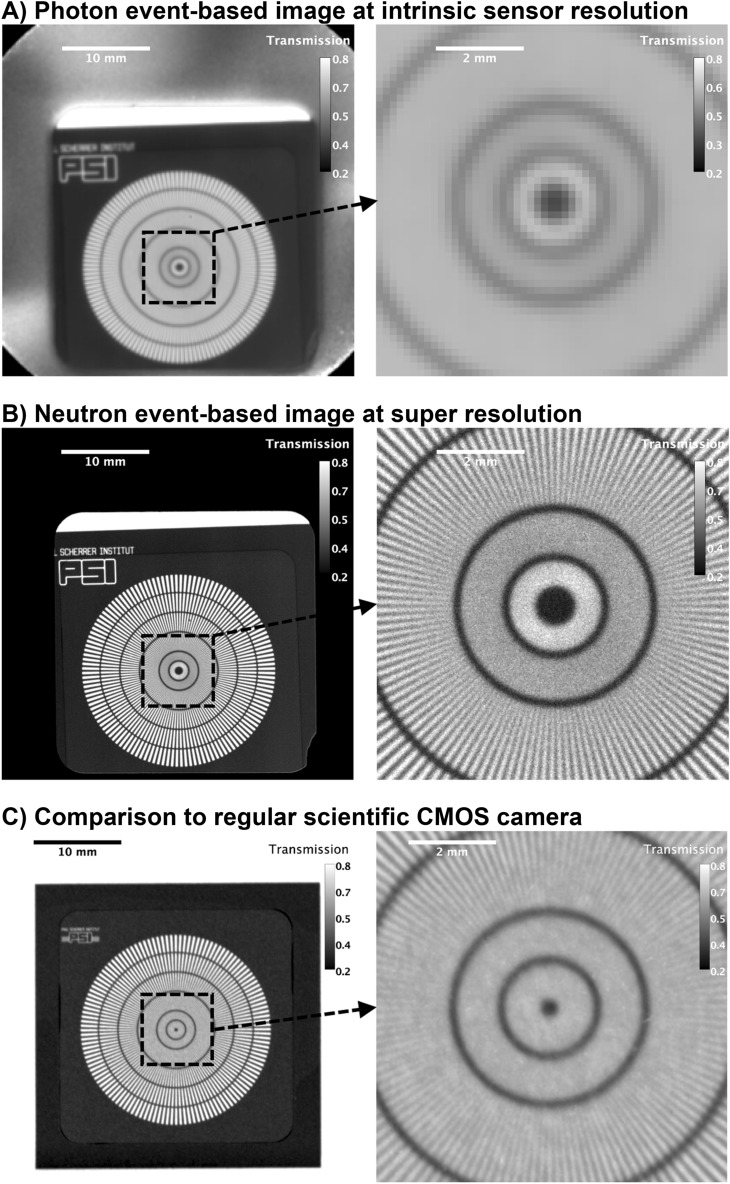
Neutron radiographs of resolution pattern. Photon event-based image recorded with TPX3Cam at intrinsic sensor resolution using 125 μm ^6^LiF:ZnS scintillator in (**A**). Neutron CoM event-based image recorded with TPX3Cam at sub-pixel sensor resolution of 0.1 × pixel-pitch using 125 μm ^6^LiF:ZnS scintillator and TPX3Cam in (**B**). For comparison, regular neutron radiograph recorded with scientific CMOS camera (ANDOR NEO) using 50 μm ^6^LiF:ZnS scintillator in (**C**).

For high spatial resolution measurements, the chopper was removed and a white-beam radiograph of a test pattern (Siemens star made from Gd^29^) with an active FoV of 4** × **4 cm^2^, as shown in Fig. 5, was recorded using a 125 μm ^6^LiF:ZnS scintillator at the BOA beamline. The total acquisition time was 2.2 h. Using the integrated photon event-data, a radiograph at intrinsic resolution of the sensor is shown in Fig. 5A. Inspecting the line-pairs of the test pattern, a resolution close to the intrinsic resolution of the sensor for that FoV with ~ 3 lp/mm or ~ 166.7 μm was observed. For CoM event-based neutron image reconstruction the resolution improved to ~ 12 lp/mm or ~ 42 μm. This yields an increase by a factor of 4 in spatial resolution comparing photon event-data at intrinsic sensor resolution with the neutron event-based image reconstruction. Inspecting the regions around the scintillator (rounded square region of 3** × **3 cm^2^ with one edge broken-off), it is noticeable that essentially no counts were measured in the active FoV that was not covered by the scintillator material. For the computation of transmission values in these regions, this resulted in a division by zero. However, to visualize this, the values were set to zero, as can be observed by the black background in Fig. 5B surrounding the scintillator. In contrast, Fig. 5A shows transmission values around 0.5 in these regions, resulting from the division of the photon background (reflected photons within the scintillator box, see Fig. 5A) with and without the sample in the beam. As discussed previously, this background is similar to the afterglow scenario in the sense of randomized single-photons that do not contribute to photon clusters, but still make their way to the detector. In the data processing, these events are discarded. The result is that light reflections are essentially eliminated and do not impact the computed transmission values. Finally, Fig. 5C shows a neutron radiograph of the resolution grating measured using a scientific CMOS camera (Andor NEO) with a 50 μm ^6^LiF:ZnS scintillator at the ANTARES beamline at the Research Neutron Source Heinz Maier-Leibnitz (FRM II)^30^. It can be observed that the spatial resolution of the radiograph is about that of the scintillator thickness, here ~ 10 lp/mm or ~ 50 μm. In comparison, the neutron event-mode image in Fig. 5B shows a 3.125 higher spatial resolution compared to that of the scintillator thickness, providing direct evidence that the limitation in scintillator thickness can be overcome with the here presented neutron event-mode imaging concept.

As a side note, while the acquisition time of 2.2 h seems relatively long for a radiograph that can resolve features down to 40 μm at a 4** × **4 cm^2^ FoV, the aim was to collect sufficient data for an in-depth analysis of the events on the scintillator while avoiding any saturation of the MCP or TPX3Cam. The hit-rate of the camera in this configuration was 15 Mhits/s, while the camera can handle up to 80 Mhits/s. The incoming neutron beam was collimated down to 1** × **1 cm^2^ using Cd-slits to reduce the hit-rate on the detector to be well below the saturation limit to avoid loss of photons, while the setup would have allowed to open the slits to 4** × **4 cm^2^, potentially increasing the flux by a factor of 16. At present, the Timepix4 chip^31^ is already being developed and will potentially offer an increase in hit-rate roughly by an order of magnitude compared to Timepix3^3^, with each new generation of Timepix chip presumably increasing the maximum hit-rate further.

## Summary and conclusions

We have demonstrated that using an event-based imaging system, it is possible to observe single spots of light on a scintillator screen down to the emission of single photons, which ultimately allows to discriminate individual particle interactions, resulting in a counting type detector principle. This capability decouples the resulting detector spatial resolution from the scintillator thickness (consequently enabling higher detection efficiency) since the position of particles interacting with the scintillator can be calculated more accurately compared to an integrated response of activated pixels. Moreover, images are not recorded like photographs by integrating charge deposited on a sensor over time, but by counting single events via the coincidence of photons detected in close proximity in space and time, significantly improving spatial resolution and reducing the noise and background of the imaging system*.* With the first measurements yielding an improvement in spatial resolution for neutron imaging of more than a factor of 3 and increased S/N by a factor of 7.5, it is assumed that by optimizing the data processing parameters, such as event-size in time and space and the potential of adding an event-shape analysis, the resolution can be increased even further. Practical limitations for this concept lie in reducing the effective pixel-pitch into smaller sub-pixels and therefore lower total number of counts thereof to a point where the resolution is limited by the counting statistics for each sub-pixel.

Using the two-stage event-based imaging concept, i.e. photon detection via clusters of pixels and neutron detection via clusters of photons, we successfully applied this technique to thermal neutron radiography with real-time data processing. The applications of this new concept are manifold even beyond neutron imaging. For example, medical imaging heavily relies on the capability of resolving features in radiographs to e.g. observe a hairline crack in a fractured bone. Utilizing the here proposed detector concept, new generation scintillation-based X-ray imaging systems that have implemented single event mode would allow to push the spatial resolution while also reducing exposure time with improved signal-to-noise ratio and therefore improving the quality of the images at a reduced radiation dose to the patient. A closer topic to the efforts presented here is material testing, whereby this concept would further close the gaps in mesoscale sciences. For example, the spatial resolution in neutron radiographs could be potentially pushed to micrometers for thermal neutron imaging, essentially providing access to microstructural features, similar to X-ray micro-CT^32,33^. Imaging with fast neutrons specifically, which has been a niche technique for very specific applications due partly to the limited achievable resolution^34^, will receive a substantial boost in applicability, potentially already through the results of this work. Utilizing the improved performance of the new detector concept, we envision it might quickly become a more routinely used imaging system for e.g. ToF neutron imaging or any sort of stroboscopic imaging measurements that require a high temporal and spatial resolution. While the results of this work show improved spatial resolution and S/N for thermal neutron imaging and an overview of the capabilities of the system, work already in progress that will be reported at a later time will include an event shape/decay and detection efficiency analysis of the system using different scintillator and lens configurations.

## Methods

The data of the TPX3Cam is recorded in a 64-bit data format for each event on the sensor (single pixel event or hit), containing the ToA, ToT, x and y coordinate of the pixel in list form^3^. To avoid creating large files, individual acquisitions were limited to 20 s each. For long acquisitions, the 20 s acquisition was executed repeatedly to reach the desired statistics. Each 20 s data-block, with a size of up to ~ 3 GB depending on the flux of visible light (or photons), was temporarily saved onto a random access memory (RAM) disc on the PC and consequently processed using straightforward CoM calculations. In the case of ToF measurements, the t_0_ signal of the chopper was fed into the camera. During the data acquisition, trigger signals are recorded as trigger-events with a timestamp. Based on the readout of the chip, the incoming events may not be in chronological order. Therefore, the event-list of the acquired data-blocks was first sorted chronologically. After sorting, the list was parsed to identify single photons (clusters of activated neighboring pixels).

With a chronological order, the identification of photon events becomes trivial since each photon event will appear as a continuous block of activated single pixels in the list. Setting a limit in x and y coordinate deviation from the first activated pixel of each photon event (30 pixels for this work), as well as a limit in time deviation (1 ms for this work), individual photon events were identified and the CoM for each interaction in x and y coordinates, as well as average ToA, was computed with the weighting in position based on the ToT (proportional to light output registered by each pixel). Single pixel events, attributed to detector noise, and clusters larger than 200 pixels, attributed to particles directly interacting with the sensor rather than the image intensifier (also known as gamma spots), were discarded in this process. Despite the relatively large acceptance in the deviation in space and time for the events, individual photons were detected with little errors, such as for example neighboring photon events being detected as one. Considering a random location for two consecutive photon events, the probability of two events being within a 30 pixel search radius in x and in y coordinate can be roughly calculated by using the search radius relative to the size of the chip in pixels squared: (30/256)^2^ = 0.014. Each detected photon-event was then saved in list form, with CoM in x and y coordinate weighted by the ToT, average ToA, total number of pixels and integrated ToT, each saved in 32-bit float format, resulting in a total of 20 Byte for each photon event entry.

For measurements using the chopper, the average ToA was saved relative to the t_0_ signal. The initial data containing the single pixel information stored on the RAM disc was deleted after the photon event list was created and subsequently saved on a large (14 TB) non-volatile memory express solid-state drive. In a final step, for neutron-event counting, the individual photon-events were parsed to find clusters of photons within a 10 pixel search radius for the 15** × **15 cm^2^ FoV and a 2 pixel search radius for the 40** × **40 mm^2^ FoV, a limit of 100 μs between individual photons and a limit for the total duration of neutron events of 500 μs was set to discriminate between individual neutron interactions. These parameters were determined from ToF measurements using the double crystal monochromator setup at BOA^21^, which allowed to observe the scintillator response as a function of time.

Finally, images were constructed from the event list by defining the effective pixel pitch (or resolution of the image), whereby the grey-value of each pixel represents the number of events (photons or neutrons, depending on what was computed) measured within the bounds of the sub-pixel. Additionally, for ToF measurements, the time information was defined in form of an image sequence, with each image representing a time-bin, set to 25 μs for this work. All images were stored in raw 32-bit unsigned data format.

## Supplementary Information

Supplementary Information.
