# Supplementary material for: New perspectives for neutron imaging through advanced event-mode data acquisition
Source: Sci Rep. 2021 Nov 1;11:21360. doi: 10.1038/s41598-021-00822-5 (PMC8560941; doi:10.1038/s41598-021-00822-5)
Supplement: Supplementary file 1 — Supplementary Information. [file 41598_2021_822_MOESM1_ESM.docx]

**­­­­**

**New Perspectives for Neutron Imaging through Advanced Event-Mode Data Acquisition**

A.S. Losko^1*^, Y. Han^1^, B. Schillinger^1^, A. Tartaglione^1^, M. Morgano^2^, M. Strobl^2^, J. Long^3^, A.S. Tremsin^4^ and M. Schulz^1^

1. *Forschungs-Neutronenquelle Heinz Maier-Leibnitz, 85748 Garching, Germany*
2. *Paul Scherrer Institute, 5232 Villigen, Switzerland*
3. *Amsterdam Scientific Instruments, 1098XG Amsterdam, Netherlands*

*4. Space Sciences Laboratory, University of California at Berkeley, Berkeley CA, 94720, USA*

*^*^Corresponding author: Tel: +49 89 289 14756 Email:* [*Adrian.losko@frm2.tum.de*](mailto:Adrian.losko@frm2.tum.de)

# Supplemental

# With the presented work on neutron imaging measurements demonstrating a new concept to radiography detectors using scintillators, supplemental information on the processes towards reaching a functional system are provided in this section.

# Photon event-mode proof of concept using visible light

Prior to measurements using neutrons, the concept of photon event-based image reconstruction was tested using visible light. For that matter, a black and white test image, shown in fig. S1 A), was printed onto a regular piece of paper with dimensions of ~10×10 cm^2^. The test image was then “photographed” within a light tight box, with visible light only being able to enter the box by penetrating the test image, essentially simulating light emitted from a scintillator. It should be noted that the printed test image was covered with two layers of black optical tape to reduce the flux of photons to a level of roughly 1.5×10^6^ s^-1^ on the sensor. The purpose for this test was to confirm the detection of single photons, since only if the events on the camera can be attributed to single photons, rather than a random light emission from the image intensifier connected to the camera, the resolution of the photon event-based image would increase. A “photograph” of the image, that is recorded similar to a regular photo-camera using the integrated response of the sensor at the native detector resolution is shown in fig. S1 B). In contrast, a photograph constructed from the CoM of individual photon events on the chip is shown in fig. S1 C). Comparing the two photographs, the improved resolution and quality of the photon event-based photograph is significantly higher as compared to that of the “regular” integrated photograph, resolving features down to ~4.2 lp/mm or a resolution of ~120 μm, an effective resolution that is ⅕ of the pixel-pitch of the sensor, agreeing with already reported results of photon event-based image reconstruction using the TPX3Cam with an image intensifier [4,5].

| 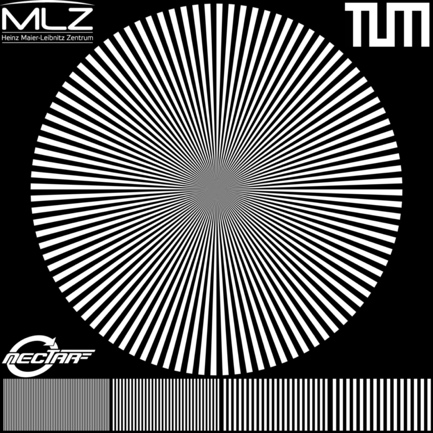 | 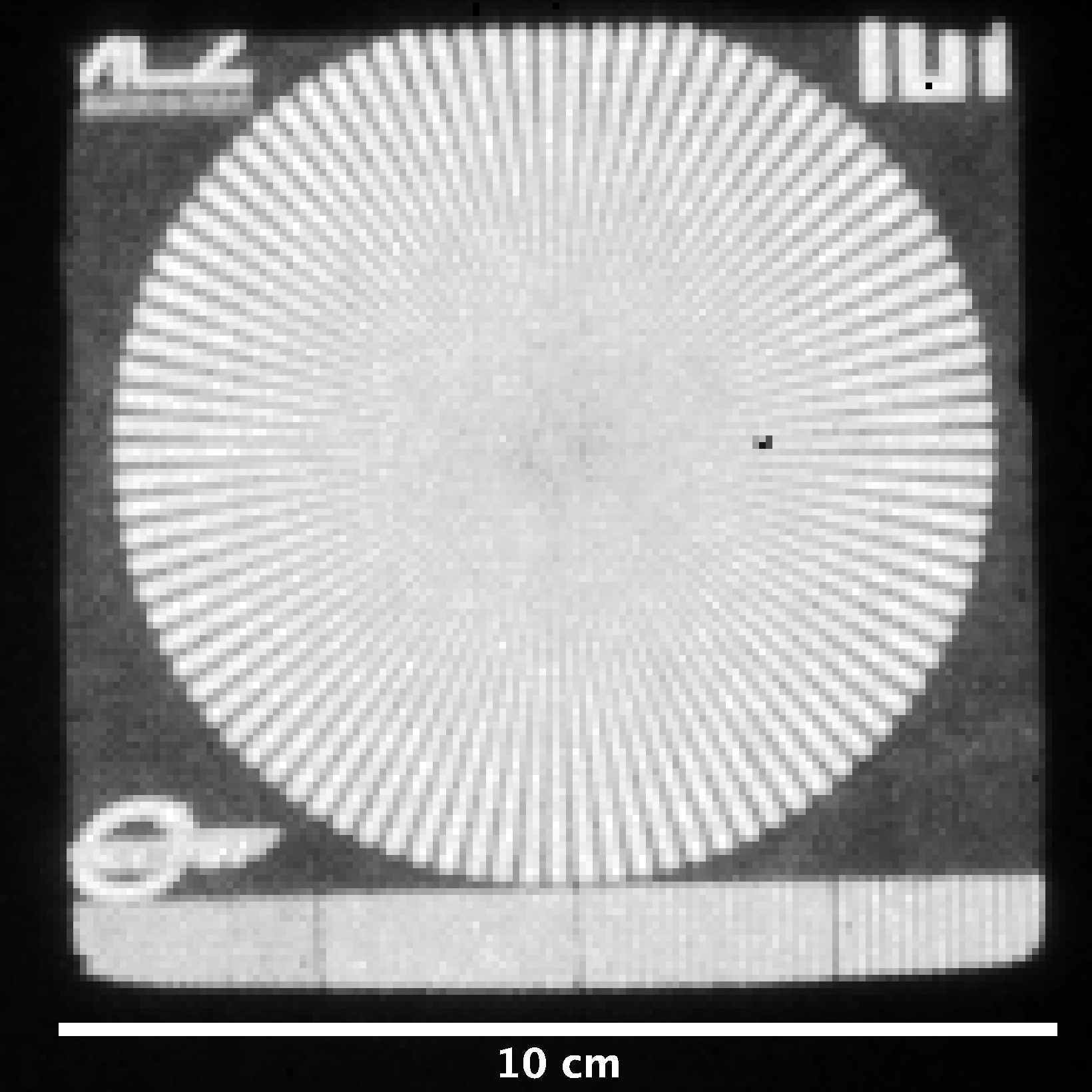 | 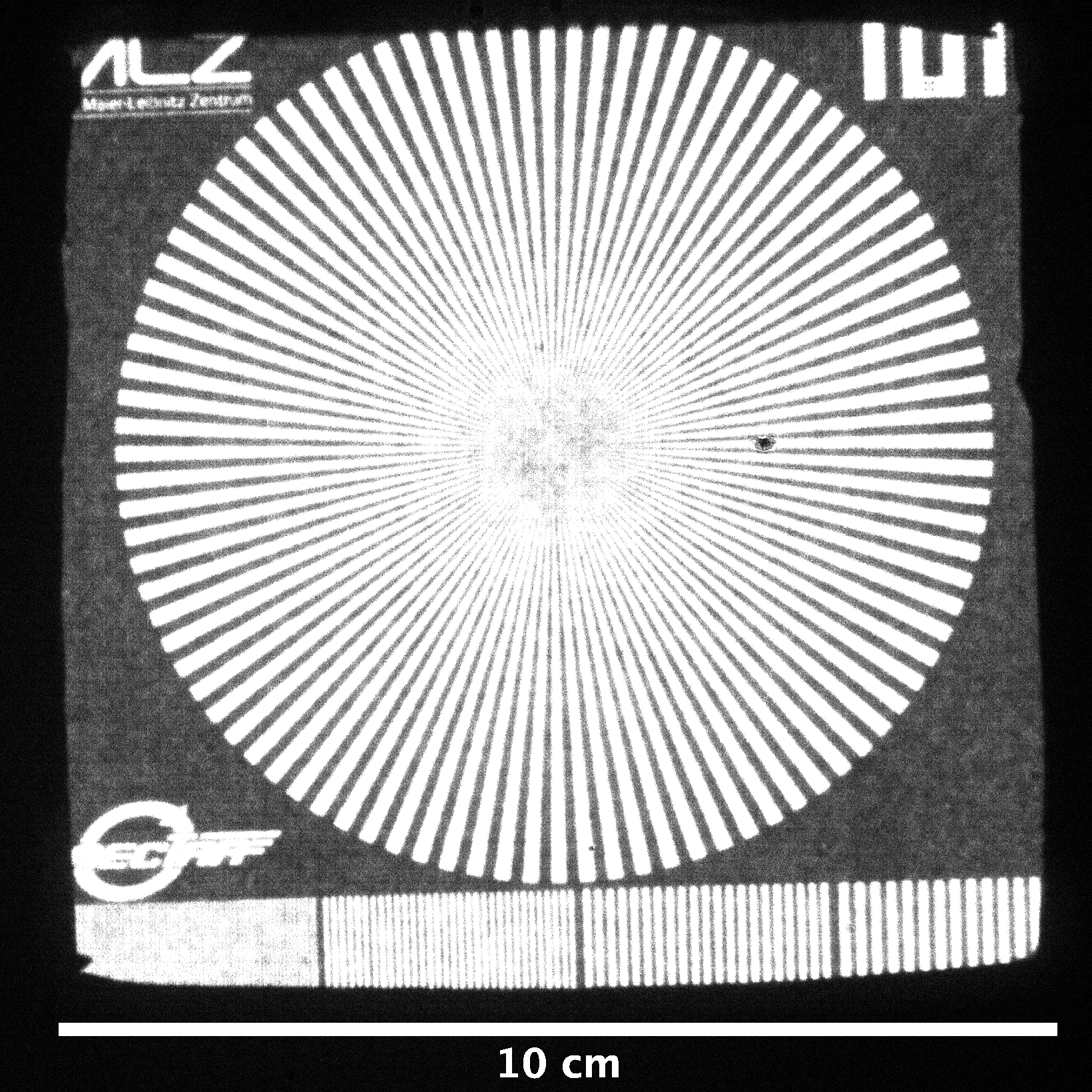 |
| --- | --- | --- |
| **Figure S1:** Test image for photon event-based proof of concept in A. Photograph using the integrated response of the camera system at native pixel resolution in B. Photograph produced from single photons *via* event-based CoM image reconstruction at sub-pixel resolution in C. | | |

# Time resolved imaging

To furthermore test and prepare software of the system for time resolved measurements, a light tight enclosure containing an incandescent 12V DC bulb was used to simulate a time-structured signal, such as in the case for ToF neutron imaging. The 12V bulb was connected to a variable DC power supply and gated by a solid-state relay. The input of the relay was connected to a signal generator operating in pulse-width modulation (PWM) mode at 20Hz and 20% modulation width, such that the bulb would be switched on for 10 ms and switched off for 40 ms. It should be noted that an incandescent bulb was used instead of a light-emitting diode, making use of the exponential increase and decrease of the light emitted in time by the bulb induced by the PWM input signal, as well as the capability of controlling the photon flux by increasing or decreasing the voltage on the lab power-supply. The light bulb was positioned in the enclosure such that only indirect light would be detected to avoid saturation of the detector. Varying the voltage supplied to the bulb that was gated by the PWM signal, the event rate was monitored and the voltage was increased until the detector reached ~15 Mhits/s (single pixel events). It was observed that at event rates close to the maximum of what the system is capable of processing (~80 Mhits/s), the detector response would deviate from a linear behavior.

Using the described setup, an image of the light flash produced by the 12V light bulb is shown in fig. S2 A). The integrated profile over the entire FoV as a function of time using photon event-based image reconstruction is shown in fig. S2 B) with the y-axis on a logarithmic scale, showing a nearly linear response of the detector by assuming an exponential response of the light bulb. Furthermore, the response shows an abrupt change in intensity at 10 ms, when the light bulb is “turned off”, owing to the high temporal resolution of the system.

| 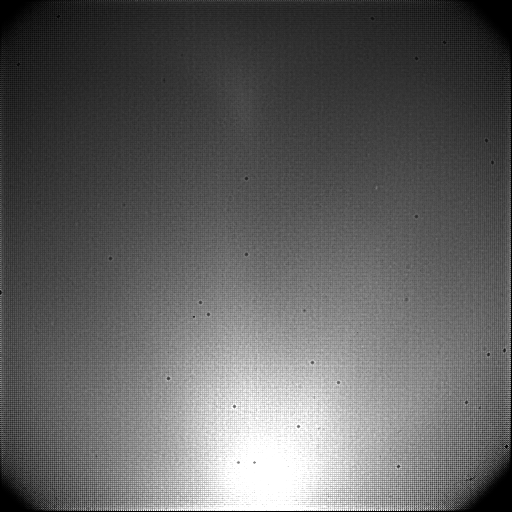 | **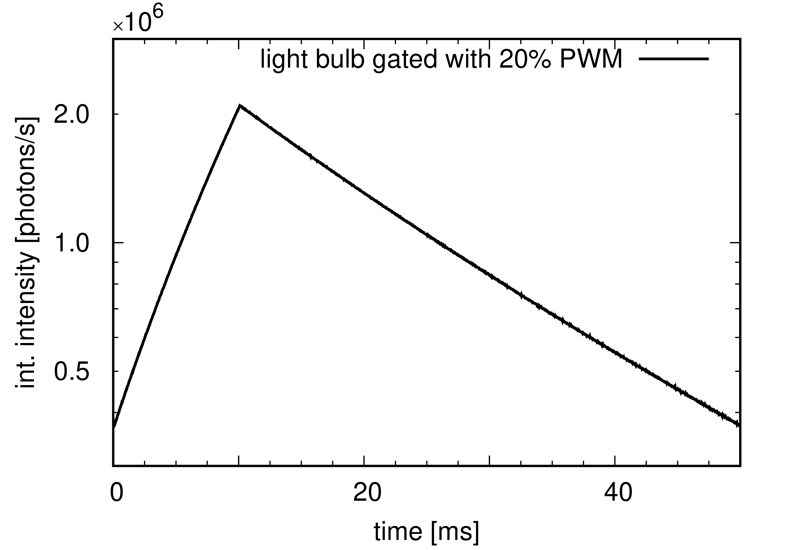** |
| --- | --- |
| **Figure S2:** Integrated image of light flashes from a 12V light bulb gated by a 20% pulse-width modulated input signal at 20Hz in A. Light-yield response thereof as a function of time integrated over the entire FoV in B. | |

# Neutron event detection and scintillator response

In a first approach to measure the decay properties of the scintillator for neutrons, measurements using the intensified TPX3Cam Optical Camera with a ^6^LiF:ZnS scintillator were performed at the BOA beamline at PSI [18]. The beamline was operating in ToF mode using a chopper spinning at 25 Hz with an ~1% wavelength resolution, $\Delta\lambda/\lambda$, at 4 Å. Furthermore, a double crystal monochromator was placed in the direct beam-path, only transmitting neutrons at 3 Å and 6 Å with the scintillator placed at 4.43 m distance to the chopper. Data were acquired in ToF mode for a total of 15 min using a ~15**×**15 cm^2^ FoV and processed in real-time for photon-event identification. The measured profile of the ^6^LiF:ZnS scintillator (50 μm thickness) response in photon event-mode is shown in fig. S3. Two peaks in the profile can be observed at ToF t = 3.28 ms and t = 6.80 ms, corresponding to the transmitted neutron wavelengths of 3 Å and 6 Å, respectively. The profile shows a sharp initial increase in intensity at the peak positions, following an exponential decay. Given that the transmission curve of the double crystal monochromator is roughly symmetric with respect to the peak position, this exponential decay can be attributed to the fluorescent lifetime of the scintillator material. Using a single exponential decay to describe the decrease in intensity, the decay constant was determined to be 0.10(9) ms. For an exponential decay, this means that after the duration of five decay constants the intensity will reduce to < 99%. This result was used to set the boundary for the duration of single neutron events to 500 μs with respect to the first observed photon. Using this boundary, as well as an acceptance radius in x and y coordinate of 10 pixels, single neutron events were observed with on average 10 photons, whereby events with less than 3 photons were discarded. With half of the photons per neutron event being emitted within one half-life, t_½_ = 76 μs, a second limit for consecutive photons being not more than 100 μs separated in time was set, furthermore reducing the probability of two consecutive neutron events detected as one.

**
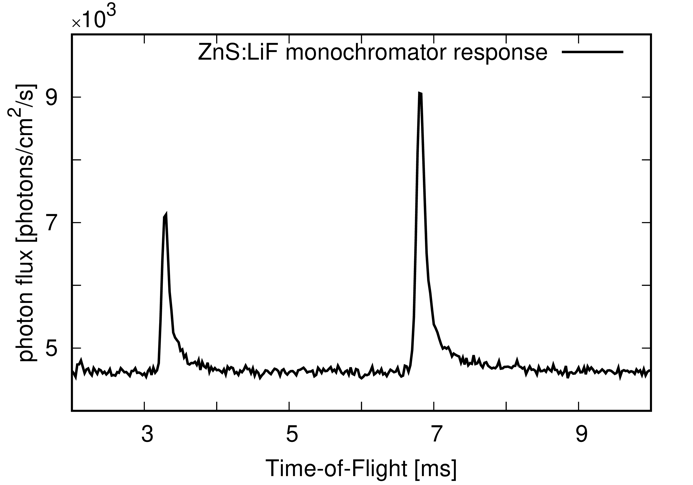
**

**Figure S3:** Detector response in photon event-mode for ^6^LiF:ZnS scintillator (50 μm thickness) using a double crystal monochromator at BOA.

# Neutron event size under the microscope for ^6^LiF:ZnS

The spatial resolution of scintillators when measured with shutter-based camera systems are typically similar to that of the scintillator thickness for scintillators which are thin compared to the particle track length, as it is the case in this work. To validate this general statement, the TPX3Cam was coupled with a zoom lens to achieve a high magnification, such that the optical resolution would surpass that of the size of the neutron events on the scintillator, allowing for direct visualization of the events. With a 5**×** magnification, resulting in a FoV equal to 2.8**×**2.8 mm^2^, the optical resolution using a Siemens star, as shown in fig. S4 A, was determined to be ~50 lp/mm or 10 μm. Using this setup, the 125 μm ^6^LiF:ZnS scintillator was imaged with a low flux of neutrons and short acquisitions of 1 ms frames (integrated raw data) without any data treatment, such that the events on the detector would be easily observable by visual inspection. The resulting image in fig. S4 B shows four captured clusters that can be attributed to four neutrons absorbed by the scintillator. The cross section along the x-axis of a single neutron event is shown in fig. S4 B on the right. The full-width-of-half-maximum using a gaussian fit to the intensities was determined to be 158 ± 12 μm, indicating that the neutron events are indeed roughly the same size as the scintillator thickness, here 125 μm.

| 1. **Optical target**   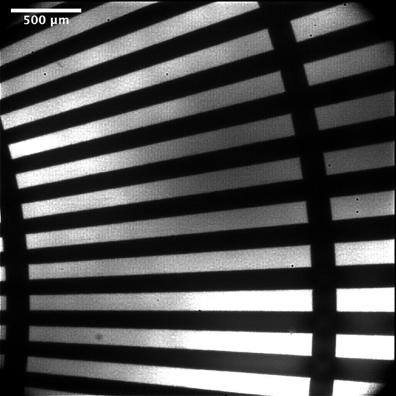 | 1. **Single neutron events on ^6^LiF:ZnS scintillator**   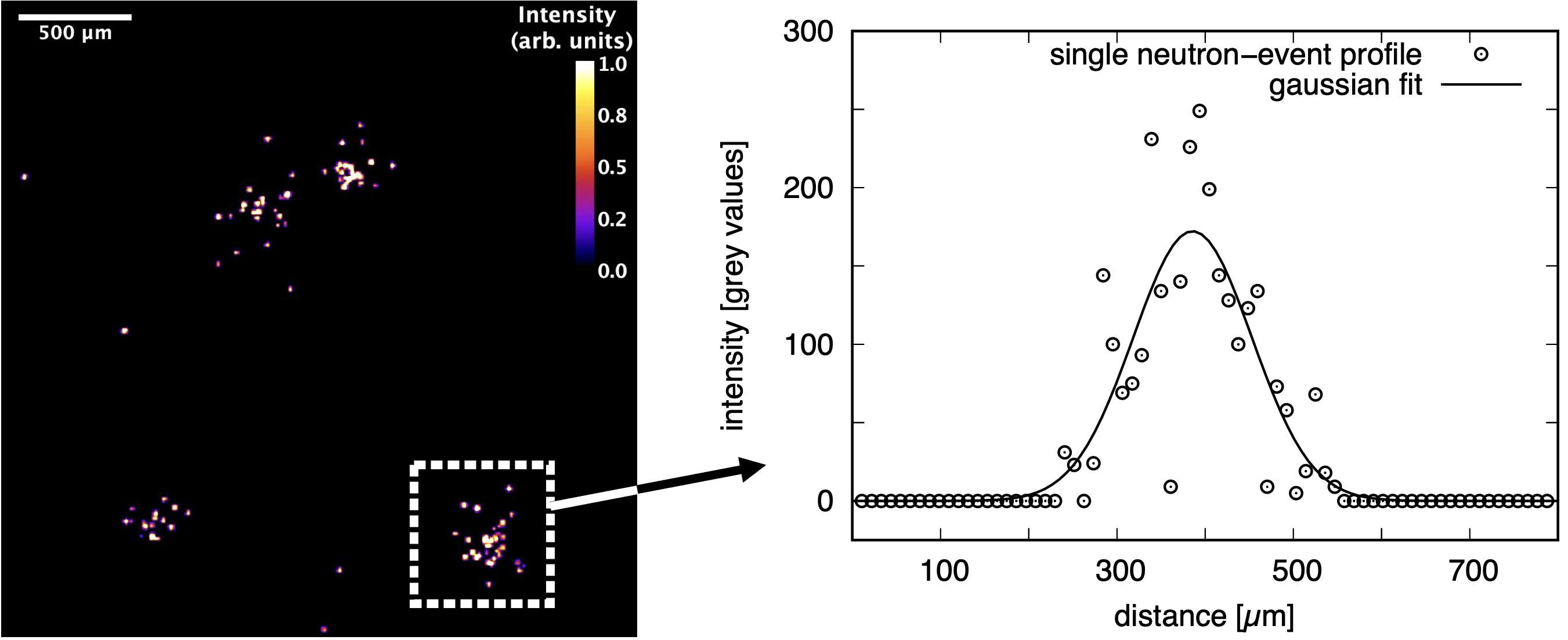 |
| --- | --- |
| **Figure S4:** Optical image of a section of a Siemens star pattern with a 2.8**×**2.8 mm^2^ FoV in A. Neutron events measured in the same configuration using a 125 μm ^6^LiF:ZnS scintillator and a 1 ms integrated raw data-frame in B, alongside a cross section plot of the event along the x-axis, including gaussian fit of the respective intensities in the plot. | |
